# Clinical characteristics of colorectal flat adenoma and the risk factors associated with missed flat adenoma: A retrospective cohort study

**DOI:** 10.1097/MD.0000000000049738

**Published:** 2026-07-10

**Authors:** Min-Kyung Yeo, Sun Hyung Kang, Hee Seok Moon, Jae Kyu Sung, Ju Seok Kim

**Affiliations:** a Department of Pathology, Chungnam National University College of Medicine, Daejeon, Korea; b Departments of Internal Medicine, Chungnam National University College of Medicine, Daejeon, Korea.

**Keywords:** colon neoplasms, colonoscopy, early detection of cancer, risk factors

## Abstract

Colorectal flat adenoma is particularly closely linked to interval cancer of the proximal colon, and therefore its clinical importance has been increasing. However, there is a lack of research on colorectal flat adenoma. This study aims to examine the clinical characteristics of colorectal flat adenoma and the risk factors related to missed flat adenoma.

This retrospective cohort study was conducted on the medical records of patients who had colonoscopies performed at this hospital (single-center, Korea) from January 2018 to December 2023. The colorectal adenomas were categorized into flat and protruding adenoma depending on their morphology, and the clinical characteristics of each group were examined. The risk factors related to the flat adenoma miss rate were analyzed for patients who had received more than 2 colonoscopies during the target research period.

Out of the 4859 patients who received index colonoscopies, 9358 cases of colorectal adenoma were discovered, of which 1734 (18.5%) were flat adenoma and 7624 (81.5%) were protruding adenoma. Multivariate analysis confirmed that males above 65 years old with a familial history of colorectal cancer and proximal location were the factors related to flat adenoma. Out of the 1304 patients who had received 2 or more colonoscopies, the 415 cases of missed flat adenoma discovered in 369 (28.3%) patients were analyzed. The flat adenoma showed a statistically significantly higher adenoma miss rate than that of the protruding adenoma group. (odds ratio 1.417; 95% confidence interval, 1.299–1.667, *P* < .001). It was also confirmed that the risk factors of missed flat adenoma were proximal location, inadequate bowel preparation, shorter withdrawal time, and concomitant protruding adenoma.

The location, bowel preparation status, withdrawal time, and presence of concomitant protruding adenoma were the factors related to missed flat adenoma. A more careful examination must be conducted when performing colonoscopies on patients who have the above risk factors.

## 1. Introduction

Colorectal cancer (CRC) is the most frequently occurring malignancy around the world and is the second highest cause of mortality.^[1,2]^ Most CRC occurs in the adenoma, and while colonoscopy examinations can both discover and remove adenoma at the same time, it is not possible to remove all adenoma during a single colonoscopy.^[3]^ There is about a 9 to 28% adenoma miss rate (AMR) being reported, and this missed adenoma (especially flat adenoma) is related to the occurrence of interval cancer.^[4]^ Therefore, in order for colonoscopies to serve as an effective screening modality, the quality of colonoscopies must be improved to reduce AMR. Up to now, the factors known to be related to AMR are bowel preparation status, withdrawal time, location of adenoma, and the skill level and experience of the doctor conducting the colonoscopy, etc, while morphology was also shown to be an important factor.^[5,6]^

According to morphology, colorectal adenoma can be broadly divided into protruding and flat adenomas, of which flat adenoma advances into severe dysplasia or carcinoma at a faster rate than protruding adenoma. Submucosal invasion is also observed more frequently, even in its early stages.^[7,8]^ Flat adenoma mainly occurs in the proximal colon, which has deep folds and tends to be small, covered with a mucus cap, and is usually morphologically flat. Therefore, during colonoscopies, the miss rate is higher than that of protruding adenoma, at 35 to 60%.^[9]^ Flat adenoma is considered a main cause of proximal colon interval cancer. To reduce the rate of missed flat adenoma, there have been attempts to use chromoendoscopy or magnifying narrow-band imaging, but these methods are still controversial.^[10]^

Also there is also a lack of research on colorectal flat adenoma, which is clinically important. There is especially sparse information on the risk factors of missed flat adenoma, which are directly related to the occurrence of interval cancer, and even the research that has been done does not show consistent results.^[11,12]^ This study aimed to characterize the clinical features of flat colorectal adenomas and to identify factors associated with their miss rate in patients undergoing repeat colonoscopy.

## 2. Methods

### 2.1. Study population

The medical records of patients who had received colonoscopies at the Chungnam National University Hospital from January 2018 to December 2023 were retrospectively analyzed. 1304 of these patients had received more than 2 colonoscopies within the 2 years of records that were analyzed, and the first colonoscopy was defined as an index colonoscopy. In order to target patients with an average risk for CRC, patients with a medical history of CRC, patients under 40 years of age with inflammatory bowel disease or gastrointestinal bleeding, patients discovered to have more than 10 polyps, and also patients where cecal intubation had failed were excluded from the research. This study was a retrospective study that only involved a review of the patients’ medical charts and was exempted from ethical approval according to the research committee regulations of our institution.

### 2.2. Colonoscopy and cofactors

All colonoscopies were conducted by gastroenterologists using colonoscopy (Olympus). Polyethylene glycol was used as the bowel preparation agent, and because all colonoscopies at this hospital were conducted in the afternoon, the preparation agent was administered as a same-day regimen. The states of cleanliness of the colon were categorized as follows, according to the Aronchick scale based on the endoscopy records of doctors who had conducted the colonoscopies.^[13]^ Excellent, > 95% of the surface is seen; good, large volume of clear liquid covering 5 to 25% of the surface, but > 90% of the surface is seen; fair: some semisolid stool that could be suctioned, but > 90% of the surface is seen; poor: semisolid stool that could not be suctioned and < 90% of the surface is seen. Two subgroups were defined as an adequate preparation group for excellent and good bowel preparation and an inadequate preparation group for fair and poor bowel preparation.

Based on the endoscopy record and pathology results, the patients were categorized according to pathology results and the size, location, and shape of the adenoma. According to the location of the adenoma, it was categorized into proximal colon (cecum, ascending colon, and hepatic flexure) and distal colon. According to the Japanese Research for Cancer of Colon and Rectum Classification, the patient cases were morphologically categorized into protruding and flat (height < half of the lesion in diameter).^[3]^ For the morphological categorization, which is the most critical factor taken into consideration in this research, the images saved to the picture archiving communications system were reviewed one more time to increase accuracy. A pathology doctor categorized the patients pathologically based on the World Health Organization and Vienna criteria for colorectal adenoma. Cases where the size of the adenoma was > 1 cm or cases where there was a histological villous or high-grade dysplasia component were defined as advanced adenoma.

### 2.3. AMRs

All cases of adenoma discovered in index colonoscopies and repeat colonoscopies were analyzed. All adenomas that were not detected during index colonoscopies but detected during repeat colonoscopies were defined as missed adenomas. “Per-adenoma” AMR was calculated by dividing the total number of adenomas discovered in repeat colonoscopies by the total number of adenomas, and the “Per-patient” AMR was analyzed by divided the number of patients with 1 or more adenomas found in repeat colonoscopy by the number of patients. Using the same method, the “Per-adenoma” AMR and the “Per-patient” AMR for flat adenoma were also analyzed.

### 2.4. Statistical analysis

Continuous variables were indicated using means and standard deviation (SD), and categorical variables were indicated using frequencies and percentages. Missing values were excluded from the analysis. The AMR and 95% confidence interval (CI) were calculated after dividing the patients into subgroups according to the shape of the adenoma, and each categorical variable was compared using Fisher exact test. The characteristics of flat colorectal adenoma were analyzed, and to check the risk factor of flat adenoma, *P* values ≤ .1 for univariate analysis were included in the multivariate models. A logistic regression was used to measure the AMR and 95% CI in the multivariate analysis and the odds ratio (OR) according to the shape of the adenoma in the multivariate analysis. All analyses were performed using SPSS software, version 20.0 (SPSS Inc.), and a *P* value (2-sided) of < .05 was considered to be significant.

## 3. Results

### 3.1. Patients’ characteristics

**Figure 1.**
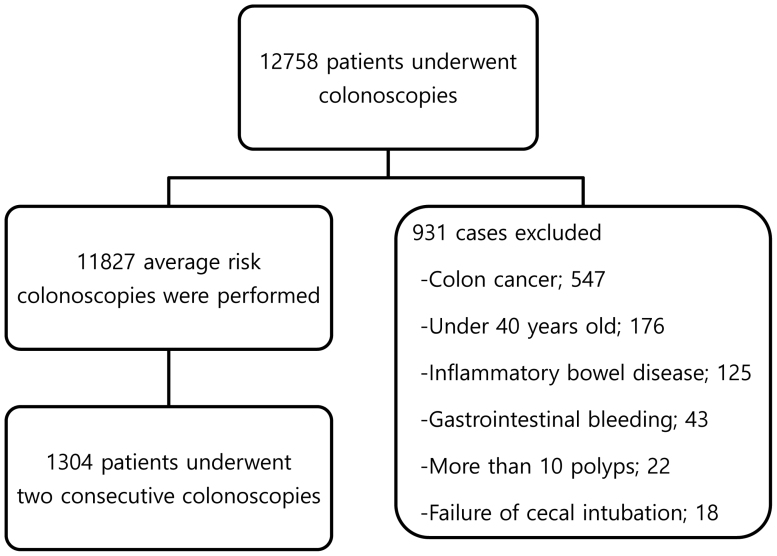
Flow chart of this study and patient analysis.

Excluding 931 patients that did not satisfy the inclusion criteria, a total of 11,827 patients were enrolled in this research. Amongst these patients, 1304 (11.0%) had received more than 2 colonoscopies (Fig. 1). Because all colonoscopies were conducted by expert gastroenterologists, the cecal intubation failure rate was very low (0.14%) 59.1% of all patients were male, and the mean age (SD) was 61.43 (8.7) years old. Out of the patients, 4859 were discovered to have adenoma, and the patients were analyzed by being categorized into 3 groups according to the morphology of the adenoma (Table 1) There were 481 patients (9.9%) in the flat adenoma group, 837 patients (17.2%) in the flat and protruding adenoma group, and 3541 patients (72.9%) in the protruding adenoma group. While there were significant differences in the sex, age, family history of CRC, and bowel preparation status of the patients in the 3 groups, there were no differences in smoking status and body mass index (BMI). With the exception of family history of CRC (25.9%), smoking (13.8%), and BMI (9.5%), there were no missing values.

**Table 1:** Clinical characteristics of patients according to the type of colorectal adenomas.

Characteristics,	Total	Flat adenoma	Flat and protruding adenoma	Protruding adenoma	*P* value†
N (%)	4859 (100)	481 (9.9)	837 (17.2)	3541 (72.9)	
Sex					< .001
Male	2857 (59.2)	276 (57.4)	623 (74.4)	1976 (55.8)	
Female	1984 (40.8)	205 (42.6)	214 (25.6)	1565 (44.2)	
Age (mean, SD)	60.43 (9.58)	62.73 (9.63)	64.99 (9.91)	62.39 (9.77)	< .001
Family History of CRC					< .001
Yes	406 (11.3)	97 (24.1)	109 (17.6)	200 (7.8)	
No	3193 (88.7)	306 (75.9)	511 (82.3)	2376 (92.2)	
Smoking					.418
Yes	673 (16.1)	72 (16.9)	131 (16.8)	470 (15.7)	
Never	3514 (83.9)	352 (83.1)	647 (83.2)	2515 (84.3)	
BMI					.585
< 23	1125 (25.6)	120 (25.9)	198 (26.2)	807 (25.4)	
≥ 23–< 25	1078 (24.5)	116 (25.0)	157 (20.7)	805 (25.3)	
≥ 25	2194 (49.9)	228 (49.1)	402 (53.1)	1564 (49.3)	
Bowel preparation					0.002
Adequate	3806 (78.3)	358 (74.4)	689 (82.3)	2759 (77.9)	
Inadequate	1053 (21.7)	123 (25.6)	148 (17.7)	782 (22.1)	

BMI = body mass index, CRC = colorectal cancer, N/n = number of participants, SD *=* standard deviation.

* Missing (n, %): Family History of CRC (1260, 25.9), Smoking (672, 13.8), BMI (462, 9.5).

† Chi-square test compares type of colorectal adenomas categories for each characteristic.

### 3.2. Detected adenoma analysis

A total of 9358 cases of adenoma were discovered in 4859 patients through index colonoscopies. There were 1734 cases of flat adenoma (18.5%) and 7624 cases of protruding adenoma (81.5%). (Table 2) 58.9% of patients were male, and the mean age (SD) was 63.61 (10.2) years old. There were missing values for family history of CRC (25.1%), smoking (13.6%), and BMI (9.4%). There were more males who were older with a family history of CRC, and in many cases, the size of the adenoma was below 6mm in the flat adenoma group compared to the protruding adenoma group. There was no significant difference depending on whether the patient smoked, their BMI, or whether the patient had a case of advanced adenoma. Multivariate analysis was conducted by adjusting for sex, age, family history of CRC, location, and size, which showed significant differences in the 2 groups. Male (OR 1.546; 95% CI, 1.191–2.008), above the age of 65 (OR 1.355; 95% CI, 1.218–1.879), family history of CRC (OR 3.137; 95% CI, 2.506–4.042), and proximal colon (OR 2.228; 95% CI, 1.870–2.805) were shown to be significantly higher in the flat adenoma group than in the protruding adenoma group, while adenoma size did not show a significant difference.

**Table 2:** “Per-adenoma” characteristics according to the type of colorectal adenomas.

Characteristics,	Total	Flat adenoma	Protruding adenoma	Univariate analysis	Multivariate analysis	
N (%)	9358 (100)	1734 (18.5)	7624 (81.5)	*P* value	OR (95% CI)	*P* value†
Sex				.000		
Female	3846 (41.1)	552 (31.8)	3294 (43.2)		1	
Male	5512 (58.9)	1182 (68.2)	4330 (56.8)		1.546 (1.191–2.008)	.001
Age (yrs)				.000		
< 65	5137 (54.9)	832 (48.0)	4305 (56.5)		1	
≥ 65	4221 (45.1)	902 (52.0)	3319 (43.5)		1.355 (1.218–1.879)	.001
Family History of CRC				.000		
No	6149 (87.6)	1005 (77.4)	5144 (89.9)		1	
Yes	870 (12.4)	293 (22.6)	577 (10.1)		3.137 (2.506–4.042)	< .001
Smoking				.928		
Yes	1358 (16.8)	261 (16.9)	1097 (16.8)			
Never	6728 (83.2)	1283 (83.1)	5445 (83.2)			
BMI				.514		
< 23	2171 (25.6)	409 (26.1)	1776 (25.7)			
≥ 23 to < 25	2026 (23.9)	358 (22.8)	1624 (23.5)			
≥ 25	4282 (50.5)	801 (51.1)	3511 (50.8)			
Location				.000		
Distal colon	5569 (59.5)	751 (43.3)	4818 (63.1)		1	
Proximal colon	3789 (40.5)	983 (56.7)	2806 (36.9)		2.228 (1.870–2.805)	< .001
Size (mm)				.027		
≥ 10	533 (5.7)	105 (6.1)	438 (5.7)		1	
≥ 6 to <10	2629 (28.1)	428 (24.6)	2201 (28.9)		0.872 (0.571–1.125)	.161
< 6	6196 (66.2)	1201 (69.3)	4985 (65.4)		1.063 (0.838–1.279)	.782
Advanced adenoma	636 (6.8)	110 (6.4)	526 (6.9)	.437		

BMI = body mass index, CI = confidence interval, CRC = colorectal cancer, N/n = number of participants, OR = odds ratio.

* Missing (n, %): Family History of CRC (2339, 25.1), Smoking (1272, 13.6), BMI (879, 9.4).

† Adjusted for sex, age, family history of CRC, location and size.

### 3.3. Missed adenoma analysis

Cases of missed adenoma were analyzed for the 1304 patients that had received more than 2 colonoscopies. The overall “Per-patient” AMR was 25.2%, and the subgroup analysis showed 28.3% (369/1304) for “Per-patient” flat AMR and 24.4% (1067/4378) for protruding AMR. The “Per-patient” miss rate of the flat adenoma was shown to be higher than of the protruding group. (OR 1.149; 95% CI, 1.003–1.316, *P* = .045) Also the overall “Per-adenoma” AMR was 17.7%. The “Per-adenoma” flat AMR was 23.9% (415/1734) and the protruding AMR was 16.3% (1240/7624). The “Per-adenoma” miss rate of the flat adenoma group was shown to have a significant difference compared to that of the protruding group. (OR 1.417; 95% CI, 1.299–1.667, *P* < .001) A total of 134 cases of flat advanced adenoma were discovered in the index and repeat colonoscopies, of which 24 cases were missed flat advanced adenoma. The “Per-patient” and “Per-adenoma” flat advanced AMR were 1.7%, and 1.4%, respectively.

### 3.4. Missed flat adenoma and risk factors

According to whether or not flat adenoma was discovered, the patients were divided into 2 categories: missed flat adenoma and diagnosed flat adenoma, and the characteristics for each of the categories were compared (Table 3). The flat adenoma discovered during the index colonoscopy had 1734 patients, and missed flat adenoma had 415 patients. 67.5% were males, and the mean age (SD) was 64.12 (11.8) years old. Compared to the diagnosed flat adenoma patients, the missed flat adenoma patients showed characteristics of older age, proximal colon location, inadequate bowel preparation status, shorter withdrawal time, and concomitant protruding adenoma during the index colonoscopy and were also statistically more significant. On the other hand, there was no difference in terms of sex, family history of CRC, BMI, adenoma size, and advanced adenoma.

**Table 3:** “Per-adenoma” characteristics of missed flat colorectal adenomas.

Characteristics,	Total	Diagnosed flat adenoma	Missed flat adenoma	*P* value
N (%)	2149 (100)	1734 (80.7)	415 (19.3)	
Sex				.179
Male	1450 (67.5)	1182 (68.2)	268 (64.6)	
Female	699 (32.5)	552 (31.8)	147 (35.4)	
Age (yrs)				.024
< 65	1005 (46.8)	832 (48.0)	173 (41.7)	
≥ 65	1145 (53.2)	902 (52.0)	242 (58.3)	
Family History of CRC				.380
No	1211 (76.9)	1005 (77.4)	206 (75.2)	
Yes	361 (23.1)	293 (22.6)	68 (24.8)	
BMI				.603
< 23	502 (25.9)	409 (26.1)	93 (24.9)	
≥ 23 to < 25	441 (22.7)	358 (22.8)	83 (22.2)	
≥ 25	999 (51.4)	801 (51.1)	198 (52.9)	
Location				.012
Distal colon	902 (42.0)	751 (43.3)	151 (36.4)	
Proximal colon	1247 (58.0)	983 (56.7)	264 (63.6)	
Size (mm)				.128
≥ 10	127 (6.0)	105 (6.1)	22 (5.3)	
≥ 6 to < 10	518 (24.1)	428 (24.6)	90 (21.8)	
< 6	1504 (69.9)	1201 (69.3)	303 (72.9)	
Bowel preparation				< .001
Adequate	1535 (71.4)	1310 (75.6)	225 (54.3)	
Fair	426 (19.8)	319 (18.4)	107 (25.8)	
Poor	188 (8.8)	105 (6.0)	83 (19.9)	
Withdrawal time (min)				< .001
≥ 6	1403 (65.3)	1167 (67.3)	236 (56.9)	
< 6	746 (34.7)	567 (32.7)	179 (43.1)	
Concomitant protruding adenoma at index colonoscopy	403 (18.7)	267 (15.4)	136 (32.7)	< .001
Advanced adenoma	134 (6.2)	110 (6.4)	24 (5.9)	.755

BMI = body mass index, CRC = colorectal cancer, N/n = number of participants.

* Missing (n, %): Family History of CRC (577, 26.8), BMI (207, 9.6).

To confirm the risk factors of missed flat adenoma, age, location, bowel preparation status, withdrawal time, and concomitant protruding adenoma, which were shown to be significant in the univariate analysis, were adjusted before conducting multivariate analysis (Table 4). Compared to the diagnosed flat adenoma group, the missed flat adenoma group showed characteristics of proximal colon location (OR 1.307; 95% CI, 1.056–1.669), inadequate bowel preparation status (OR 2.509; 95% CI, 1.977–3.178), a withdrawal time of under 6 minutes (OR 1.551; 95% CI, 1.237–1.944), and concomitant protruding adenoma at index colonoscopy (OR 2.587; 95% CI, 1.985–3.310), while in terms of age, the 2 groups showed no difference. For the subgroup analysis, inadequate bowel preparation was divided into fair and poor preparation for analysis. Compared to the adequate preparation group, both the fair (OR 1.943; 95% CI, 1.482–2.545) and the poor preparation group (OR 4.592; 95% CI, 3.288–6.413) were shown to have significantly more cases of missed flat adenoma.

**Table 4:** Multivariate analysis result of missed flat colorectal adenomas.

Variables	OR (95% CI)	*P* value*
Age (yrs)		
< 65	Reference (1.000)	
≥ 65	1.120 (0.923–1.392)	.119
Location		
Distal colon	Reference (1.000)	
Proximal colon	1.307 (1.056–1.669)	.036
Bowel preparation		
Adequate	Reference (1.000)	
Fair	1.943 (1.482–2.545)	< .001
Poor	4.592 (3.288–6.413)	< .001
Withdrawal time (min)		
≥ 6	Reference (1.000)	
< 6	1.551 (1.237–1.944)	.002
Concomitant protruding adenoma at index colonoscopy		
No	Reference (1.000)	
Yes	2.587 (1.985–3.310)	< .001

CI = confidence interval, OR = odds ratio.

* Adjusted for age, location, bowel preparation, withdrawal time, and concomitant protruding adenoma.

## 4. Discussion

Our study results showed that the “Per-patient” and “Per-adenoma” AMR of protruding adenoma were 24.4% and 16.3%, respectively, “Per-patient” and “Per-adenoma” AMR of flat adenoma were 28.0% and 23.9% respectively, confirming that the miss rate of flat adenoma was significantly higher. Compared to protruding adenoma, flat adenoma has a more aggressive biological characteristic, and because the occurrence of flat adenoma is increasing, its clinical significance is also increasing.^[14]^ It is known to occur more often in the Eastern Hemisphere than the Western Hemisphere,^[15]^ and even in our study, flat adenoma made up 18.5% of all adenomas. Due to its morphological characteristics, the miss rate of flat adenoma was higher than protruding adenoma. According to a meta-analysis, the overall AMR was 26% (95% CI, 23–30%), but the flat AMR was high at 34% (95% CI, 24–45%).^[16]^

The proximal location, inadequate bowel preparation, a withdrawal time of under 6 minutes, and a concomitant protruding adenoma at index colonoscopy were shown to be factors related to the miss rate of flat adenoma from the results of this research. There is also research on the relationship between age and missed flat adenoma which reports that the miss rate of flat adenoma is significantly higher in patients older than 60 years old (OR 2.062; 95% CI, 1.390–3.061).^[17]^ But while our research showed there was a tendency for a high flat AMR for patients above the age of 65 (*P* = .024), no difference was confirmed from the multivariate analysis (OR 1.120; 95% CI, 0.923–1.392). Further research must be conducted on this controversial area. For advanced adenoma, there was also no significant difference for the 2 groups (*P* = .755), and this is considered to be due to defining advanced adenoma as lesions that are larger than 1cm, when in fact there is a lower likelihood of overlooking lesions in colonoscopies the larger the size of the lesion.

The fact that the AMR changes according to the location of the flat adenoma has also been previously confirmed in other research.^[18]^ In this research, the flat AMR of the proximal colon was also significantly higher than that of the distal colon (OR 1.307; 95% CI, 1.056–1.669). The reason for this difference is due to there being more cases of flat adenoma in the proximal colon which are therefore missed, and compared to the distal colon, the proximal colon is anatomically deeper with more wrinkles, and therefore when flat adenoma occurs in this area it can be difficult to discover.^[19,20]^ Therefore there is a need to examine the proximal colon with extra care when conducting colonoscopies, and methods to achieve this include using the retroflexion technique in the ascending colon and also increasing the withdrawal time.^[21]^

Bowel preparation status was also confirmed to be closely linked with AMR.^[22]^ Because all patients who receive colonoscopies have different compliance for bowel preparation, their bowel preparation status differs, and this can affect the AMR. When the cleanliness of the intestines is not adequate, the mucus and chyme secreted by the small intestine are not adequately removed and build up in the proximal colon, which can interfere with the discovery of adenoma.^[23,24]^ Flat adenomas in the proximal colon are affected more by the cleanliness of the intestines than protruding adenomas are. In this research as well, the inadequate bowel preparation group showed a significantly higher flat AMR (OR 2.509; 95% CI, 1.977–3.178) compared to that of the adequate bowel preparation group. The subgroup analysis also showed that not only for the poor preparation group (OR 4.592; 95% CI, 3.288–6.413) but also for the fair preparation group (OR 1.943; 95% CI, 1.482–2.545), the flat AMR was significantly higher than that of the adequate preparation group. The withdrawal time was also an important factor for AMR.^[25]^ Compared to the group with a withdrawal time of more than 6 minutes, the group with a withdrawal time of < 6 minutes had a higher flat AMR (OR 1.551; 95% CI, 1.237–1.944).

There is not a lot of research on the clinical characteristics of flat adenoma. The multivariate analysis results showed that in the flat adenoma group, compared to the protruding adenoma group, there were more male patients, patients of older age, patients with a family history of CRC, and patients with proximal colon location. While there are claims that being male is a risk factor for flat adenoma, there is still controversy because there is also research that claims the risk factor of flat adenoma is greater in women.^[26,27]^ It is considered that the contradictory results are due to the differences in ethnicity and environment. Older age and a history of CRC are well-known risk factors for colorectal adenoma and invasive cancer, which also show similar tendencies in flat adenoma.^[28,29]^ In this research, it was also shown that there was a significant occurrence in the flat adenoma group for patients who were older and had a family history. The reason for the criteria of CRC screening being set as the age of the patient is based on these results. It is well-known that flat adenoma occurs frequently in the proximal colon,^[8,23]^ and the results of this research confirm this as well. While the flat adenoma group was shown to have a smaller size compared to that of the protruding adenoma group (*P* = .027), the multivariate analysis showed that there was no significant difference (OR 1.063; 95% CI, 0.838–1.279). While there is research that claims that in comparison to protruding adenoma, flat adenoma is smaller in size, this is still a controversial area of research, and there is a need for continuous research on this claim.^[6,19,30]^

The limitations of this research are as follows. First, because this was a retrospective study, the analysis was conducted based on the records left at the time of the procedure; there can be inter-observer variation. To reduce this variation, the categorization according to the morphology of adenoma, which is the most important factor in this research, was reevaluated by reviewing the images saved to picture archiving communications system. Flat adenoma can be further categorized into flat elevated and flat depressed lesions, and it is well-known that flat depressed adenoma shows a more aggressive nature.^[31]^ However, because there was a very small number of flat depressed adenomas discovered in this research (0.02%), these cases were not separately analyzed due to the possibility of selection bias. Another limitation of the retrospective study is that there are differences in the indications for colonoscopy.

In summary, 18.5% of all adenomas in this research were flat adenomas, and male sex, older age, family history of CRC, and proximal location were factors related to flat adenomas. Also, the flat AMR was significantly higher compared to that of protruding adenoma, and older age, proximal location, fair and poor bowel preparation status, shorter withdrawal time, and concomitant protruding adenoma were the risk factors for missed flat adenoma. A more careful examination and special care must be taken when conducting colonoscopies on patients that have the above risk factors.

## Author contributions

**Conceptualization:** Min-Kyung Yeo, Hee Seok Moon, Ju Seok Kim.

**Data curation:** Min-Kyung Yeo, Jae Kyu Sung.

**Investigation:** Min-Kyung Yeo.

**Formal analysis:** Sun Hyung Kang.

**Methodology:** Sun Hyung Kang, Ju Seok Kim.

**Project administration:** Hee Seok Moon.

**Resources:** Jae Kyu Sung.

**Software:** Hee Seok Moon.

**Supervision:** Ju Seok Kim.

**Validation:** Ju Seok Kim.

**Visualization:** Jae Kyu Sung.

**Writing** – **original draft:** Min-Kyung Yeo.

**Writing** – **review & editing:** Ju Seok Kim.
